# Experimental Evaluation of a Granular Damping Element

**DOI:** 10.3390/polym16101440

**Published:** 2024-05-19

**Authors:** Sanel Avdić, Marko Nagode, Jernej Klemenc, Simon Oman

**Affiliations:** Faculty of Mechanical Engineering, University of Ljubljana, Aškerčeva Cesta 6, 1000 Ljubljana, Slovenia; sanel.avdic@fs.uni-lj.si (S.A.); marko.nagode@fs.uni-lj.si (M.N.); jernej.klemenc@fs.uni-lj.si (J.K.)

**Keywords:** granular materials, polyoxymethylene, vibration damping, stiffness, lightweight design

## Abstract

Due to their advantages—longer internal force delay compared to bulk materials, resistance to harsh conditions, damping of a wide frequency spectrum, insensitivity to ambient temperature, high reliability and low cost—granular materials are seen as an opportunity for the development of high-performance, lightweight vibration-damping elements (particle dampers). The performance of particle dampers is affected by numerous parameters, such as the base material, the size of the granules, the flowability, the initial prestress, etc. In this work, a series of experiments were performed on specimens with different combinations of influencing parameters. Energy-based design parameters were used to describe the overall vibration-damping performance. The results provided information for a deeper understanding of the dissipation mechanisms and their mutual correlation, as well as the influence of different parameters (base material, granule size and flowability) on the overall damping performance. A comparison of the performance of particle dampers with carbon steel and polyoxymethylene granules and conventional rubber dampers is given. The results show that the damping performance of particle dampers can be up to 4 times higher compared to conventional bulk material-based rubber dampers, even though rubber as a material has better vibration-damping properties than the two granular materials in particle dampers. However, when additional design features such as mass and stiffness are introduced, the results show that the overall performance of particle dampers with polyoxymethylene granules can be up to 3 times higher compared to particle dampers with carbon steel granules and conventional bulk material-based rubber dampers.

## 1. Introduction

Vibrations are a common, often unavoidable phenomenon in daily life. The characterization of vibrations is usually based on their frequency and amplitude, and in general, they are divided into structural and acoustic vibrations. The study of vibrations is of great interest due to their extensive influence on various fields of science and life; therefore, vibration control is one of the major challenges in the design of structures. Damping of vibrations is typically accomplished through active damping techniques (which use sensors and actuators to detect and control vibrations in real time), passive damping techniques (where energy is dissipated through specific materials or structural components without the need for external control or energy input), or a combination of the two mentioned methods. Due to the intricate nature and paramount significance of vibrations, there are continuous advancements in the field of vibration control. Sun [1] developed an analytical model to represent the biodynamic responses of the seated human body to tri-axial translational vibrations. In a related context, Seidler [2] studied the health effects of railway-induced vibration combined with railway noise. A review on noise and vibration suppression in hybrid electric vehicles is given by Qin [3]. Zhang [4] proposed a method for designing vibration absorbers for railroad bridges. Extending the focus to the influence of environmental factors on structural vibrations, numerous researchers have explored railway-induced vibrations [5,6] or seismic-induced vibrations [7,8].

When it comes to materials used for vibration damping, they can be divided into two major groups: metal alloys, encompassing metal matrix composites, and thermoplastics, thermosets, and other polymers, including fiber-reinforced plastics and composites [9]. Traditionally, rubber-based damping systems are widely used and studied [10]. Due to their low weight and low cost, many researchers are investigating the damping properties of plant fibers [11], fiber-reinforced polymers [12,13], polymer matrix composites [13,14], and 3D printed structures [15]. Not many non-metallic materials meet the necessary stiffness requirements, and due to that, metal alloys, such as lightweight aluminum-based alloys [16], and cold-formed steel decking for commercial and residential buildings [17], continue to play a role in the development of damping elements. Metal matrix composites, such as magnesium matrix composites, have also been widely used and studied [18,19].

Although there is a wide range of applicable materials, none singularly fulfill the dual requirements of meeting current vibration-damping standards while providing adequate load-bearing properties. Metal alloys, while offering high stiffness and load-bearing capacity, often have low vibration-damping capacity. They are also heavy, which can lead to increased energy consumption and structural stress. Additionally, metal alloys can be prone to fatigue and corrosion, reducing their long-term effectiveness and requiring regular maintenance. Polymer-based damping systems, on the other hand, have high vibration-damping capacity but low stiffness. This can lead to issues with structural stability under higher loads. Polymer-based damping systems can also degrade over time due to exposure to environmental factors such as temperature and humidity, reducing their effectiveness and requiring replacement. All of this underscores the ongoing challenge in the pursuit of optimal materials for effective vibration damping and structural support.

To enhance both the stiffness and damping properties of a structure, a novel approach leveraging the impact of pressure on stiffness change and employing a combination of pressure and force chain mechanisms to enhance damping properties can be implemented. This innovative method is based on the patented “dissipative bulk and granular system technology” [20]. This method involves the use of granular material enclosed in a confined space, known as a granular damping element (particle damper). Granular materials are defined as a collection of solid particles whose deformations under shear are insignificant compared to the dislocation of the center of mass of the particles [21]. They can neither be classified as solid, liquid nor gas because of their complex behavior. A granulate with appropriate granulation properties behaves similarly to a non-Newtonian liquid, facilitating more or less uniform pressure distribution throughout its volume without being subjected to shear forces that could compromise its integrity, unlike a homogeneous polymer piece under pressure. Due to these advantageous vibration-damping properties over traditional bulk materials and tunable mechanical properties, granular materials are seen as an opportunity for the development of high-performance, lightweight vibration-damping elements (particle dampers). The efficacy of these dampers relies on two fundamental mechanisms of energy dissipation: the pressure–frequency superposition principle at the molecular scale and the physical interactions between granules at the macro scale [9].

### Motivation and Aims

The main challenge in developing mechanical structures is to incorporate all the required mechanical properties such as strength, durability, weight-to-load ratio, and vibration-damping capacity, as well as non-mechanical properties such as environmental impact and cost. Currently available materials and manufacturing technologies are often unable to meet the necessary requirements set for damping elements. This is because the above requirements are mutually exclusive—enhancements in strength and durability often come at the expense of weight-to-load ratio and vibration-damping capacity, and vice versa. Although the granular-material-related phenomena pose challenges to comprehension, they also present an opportunity to develop mechanical structures with a significantly improved equilibrium among essential mechanical properties.

The patents proposed by Emri [20,22,23,24,25,26], which explain the relationships between the molecular-scale interactions between molecules and the macroscopic interactions between granules, and the influence of these interactions on the vibration-damping and load-bearing properties of granular materials, and the earlier research by Gosar [9] were used as the basis for this research. The objective of this study is to experimentally determine the relations between certain parameters of granular materials (granule size, flowability, and base material), vibration-damping characteristics, and load-bearing capacity, as well as to compare the current state of granular-based vibration-damping technology with the conventional vibration-damping elements. The knowledge gained in this study can be used to better understand the phenomena associated with granular materials and for further development of the new generation of lightweight load-bearing granular-based damping elements.

## 2. Materials and Methods

### 2.1. Theoretical Background

#### 2.1.1. Particle Damping Technology

Particle damping technology, a passive form of vibration-damping, involves employing granular material enclosed in a container attached to the vibrating structure [27]. The damping capacity of granular damping elements is derived from a combination of different mechanisms, such as energy loss through inelastic collisions among particles and interactions with the container walls [28], momentum exchange and acoustic radiation [29]. Due to these combined mechanisms, the damping properties of granular damping elements exhibit high nonlinearity and complexity [30]. Several factors influence their damping characteristics, encompassing particle size, shape, and material [31], as well as container geometry and filling ratio [32,33]. The formation of force chains (chains of inter-particle contact forces) has a very important role in granular materials’ behavior [34]. Particle damping technology finds widespread application in various industries, notably automotive [35], aerospace [36], and railways [37]. Granular damping elements offer advantages such as resistance to harsh conditions, low cost, low mass, high reliability, and a wide range of operating frequencies. Particle damping technology also offers a compelling opportunity to address environmental concerns by utilizing materials that contribute to environmental problems, such as plastic and rubber waste [38]. However, a major drawback of current granular damping elements is that they are attached to structures as non-load-bearing components, resulting in additional weight and indirect energy loss for lightweight structures. Proper integration of granular materials throughout the system holds the potential not only to enhance vibration-damping properties but also to augment the load-bearing capacity of structures.

#### 2.1.2. Flowability

Under certain conditions, granular materials exhibit fluid-like behavior. Flowability is not an inherent material property; instead, it depends on factors such as the geometry of tested samples, surface roughness, cohesion, etc. The ability of granular materials to flow has been the subject of numerous previous studies and is of great interest to various industries, including pharmaceuticals [39], bone tissue engineering [40], geotechnical engineering [41], transportation [42], and production [43]. Flowability is a critical factor affecting the vibration-damping properties of granular materials, as it determines the ability of granules to reorganize and redistribute within a container, influencing the overall effectiveness of the material in dissipating energy.

**Figure 1:**
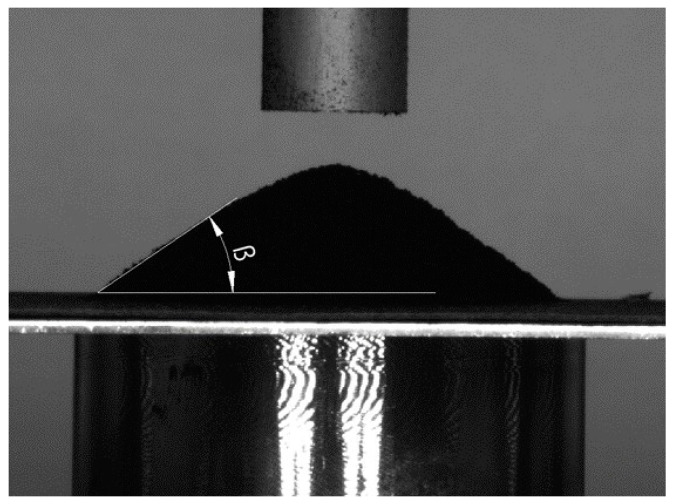
Angle of repose.

Numerous methods are used to measure flowability, such as the granular friction analyzer [44,45], hall flow meter [46], and angle of repose [47], etc. For the purpose of this study, flowability was measured by determining the angle of repose (Figure 1). The angle of repose is defined as the steepest slope of unconfined material measured from the horizontal plane on which the material can be heaped without collapsing. It can be measured by different methods, such as the tilting box method, rotating drum method, fixed funnel method, and hollow cylinder method [48]. The angle of repose is influenced by several parameters of the granules, such as size, shape, and base material. In general, a lower angle of repose value indicates better flowability.

### 2.2. Experimental Section

#### 2.2.1. Material Selection

The materials used for the experiments consist of a mixture of natural rubber (NR) and styrene-butadiene rubber (SBR), thermoplastic polyurethane (TPU), carbon steel (CS), and polyoxymethylene (POM). The reference damping element was made from a mixture of NR and SBR. TPU was selected for the fabrication of the granular material container due to its low stiffness and density, and the ability to be 3D printed using stereolithography (SLA) printing process. The low stiffness ensures high deformations and increased rearrangement of the granules, and the low density ensures a low mass of a container. CS and POM were supplied in granular form. The choice of these granular materials is primarily driven by their high accessibility and their significantly different physical properties such as density, modulus of elasticity and internal damping capacity. This selection enables a comparison of the performance of granular damping elements filled with metal and polymer granules with conventional rubber damping elements, providing valuable insight into understanding the influence of material properties on damping capacity. Table 1 provides an overview of the physical properties of CS and POM.

#### 2.2.2. Specimen Preparation

**Figure 2:**
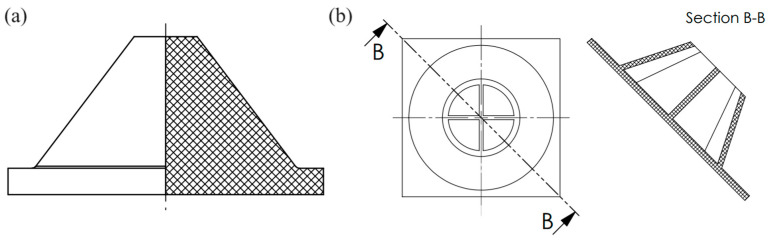
(**a**) Reference damping element, (**b**) Granular material container.

The damping element made of rubber mixture (Figure 2a) was the reference specimen (Specimen 7). It consists of a square base with a width of 60 mm and a height of 5 mm, and a cone with a base diameter of 50 mm and a top diameter of 12 mm. The height of the damping element is 30 mm.

The granular material container (Figure 2b) was fabricated using SLA 3D printing technology and consists of two distinct parts: the container body and the cover part. The base of the container body is a 60 mm wide square with a height of 2.5 mm, while the upper part takes the form of a hollow cone with a base diameter of 55 mm and a top diameter of 30 mm. The walls of the cone are 2.5 mm thick. Inside the hollow cone, two support walls in the shape of a cross, with a wall thickness of 1.5 mm, have been incorporated. The cover part of the container was manufactured separately, and it has a thickness of 2 mm. The height of the granular material container is 30 mm.

**Figure 3:**
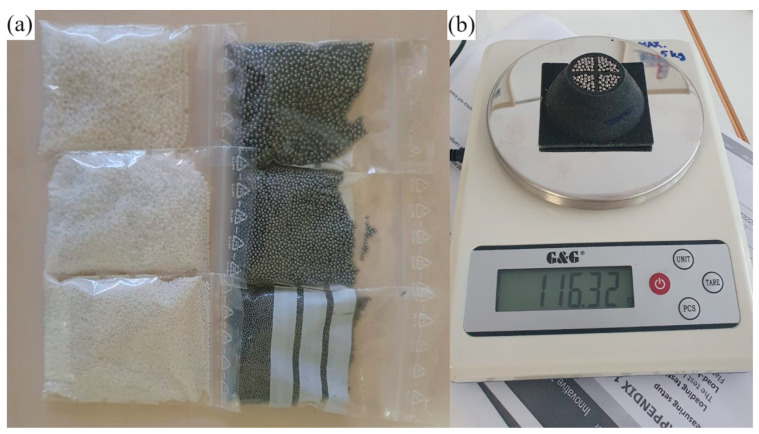
(**a**) Granular material, (**b**) specimen preparation.

The granular material used for the purpose of this study was in the form of spheres with diameters of 2 mm, 1.5 mm, and 1 mm (Figure 3a). The granular container was filled in three steps, each consisting of a first phase in which a small amount of granular material was added, followed by the compaction phase. The method of filling the granular container in this manner ensured the repeatability of the volume ratio (*κ*) defined by Equation (1), where *V_m_* represents the volume of granular material defined by the measured sample mass (*m_m_*) and material density (*ρ_m_*), and *V_0_* represents the volume of the granular material container cavity (Figure 3b).

(1) $$\kappa =\frac{V_{m}}{V_{0}},$$

Following the filling process, the container was sealed by gluing on the cover part. Six granular damping elements (Specimens 1–6) were fabricated. Table 2 provides information on the material type, size, mass, and volume ratio for each specimen.

#### 2.2.3. Testing Procedure

**Figure 4:**
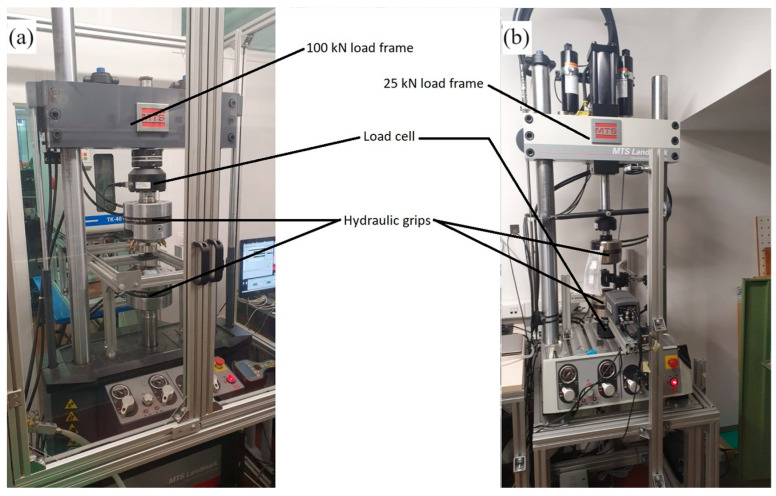
Servo-hydraulic load frames: (**a**) 100 kN load frame, (**b**) 25 kN load frame.

The experimental evaluation was conducted on the MTS Landmark servo-hydraulic system with two load frames (Figure 4). The 25 kN load frame (5 kN load cell) was used for the evaluation of the rubber damping element, while experiments involving the granular damping elements were conducted on the 100 kN load frame (1 kN load cell).

**Figure 5:**
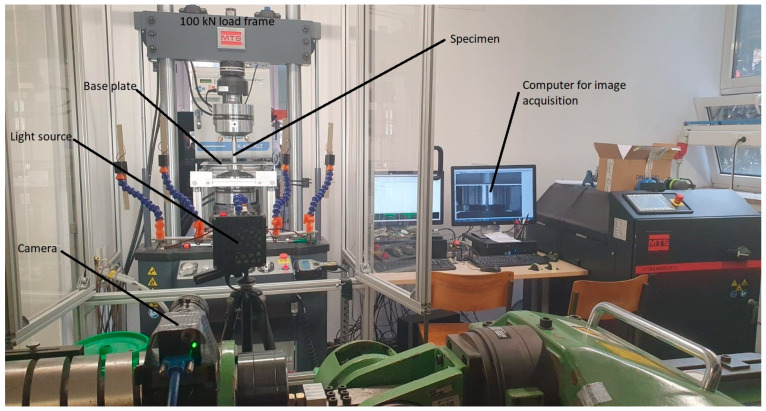
Angle of repose experiment setup.

To determine the flowability properties of granular materials, the angle of repose experiment was carried out (Figure 5). The experiment was performed by lifting the hollow cylinder specimen (inner diameter 20 mm, wall thickness 2 mm, height 100 mm) filled with a granular material at a constant velocity of 50 mm/min. After lifting the specimen, the granular material formed a heap on a base plate, which was captured by the camera. The test on each specimen was performed twice.

**Figure 6:**
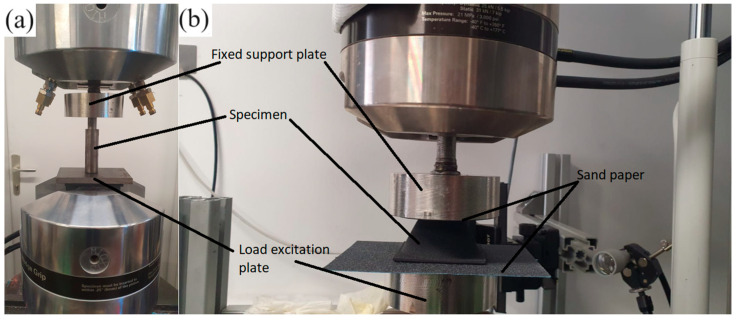
Experiment setup: (**a**) uniaxial compression, (**b**) damping element evaluation.

Uniaxial compression tests were carried out to determine the stiffness of confined granular material. A hollow cylinder specimen (inner diameter 12.5 mm, wall thickness 2 mm, height 50 mm) filled with granular material was placed between two loading plates and then compressed at a constant velocity of 10 mm/min up to a force of 500 N (Figure 6a). Throughout the tests, the mass of samples was kept constant (20 g for carbon steel, 3.4 g for POM). Force-displacement diagrams for five loading/unloading cycles were recorded for each specimen. The test on each specimen was performed twice.

During the evaluation of the damping elements, they were placed in between load plates which were covered by sandpaper (K120) to prevent sliding (Figure 6b). The evaluation was carried out in three steps. The first step involved a static test with a loading defined by a displacement velocity of 10 mm/min. The loading cycle began with a preload of 45 N and a time delay of 1 min. After 1 min, the specimens were loaded up to 450 N, and five loading/unloading cycles were performed for each specimen. The second and third steps were dynamic tests at frequencies of 0.5, 5, 10 and 20 Hz, respectively. The test started with a preload of 100 N and a 1 min time delay. After the time delay, the vibrations were applied with an amplitude of 0.25 mm. A total of 1000 loading/unloading cycles were recorded. The reason for choosing these test frequencies was to keep the amplitude of the vibrations relatively high, which implies higher loads since the main objective of the work is to demonstrate the potential of using granular damping elements also as load-bearing structures dynamically loaded with high amplitudes (dynamic factor app. 0.3). The test on each specimen was performed twice.

## 3. Results

### 3.1. Angle of Repose

**Figure A1:**
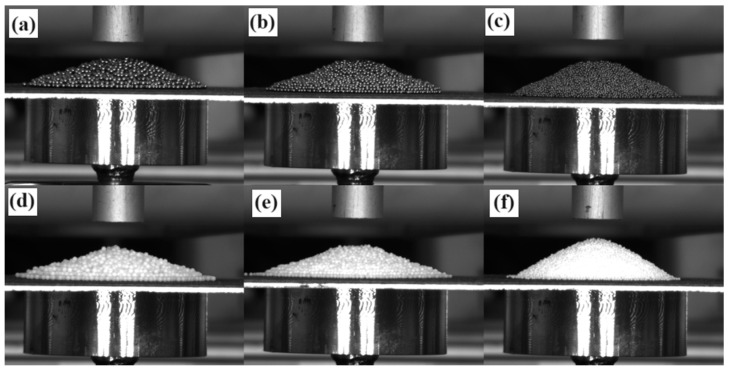
Experimental results for the angle of repose: (**a**) carbon steel, d = 2 mm, (**b**) carbon steel, d = 1.5 mm, (**c**) carbon steel, d = 1 mm, (**d**) POM, d = 2 mm, (**e**) POM, d = 1.5 mm, (**f**) POM, d = 1 mm.

The software “GNU Image Manipulation Program (GIMP 2.10.34)” was used for the postprocessing of the images acquired during the angle of repose test. During the postprocessing phase, it was found that the angles on the right and left sides of the cone were different. To address this discrepancy, the average value of the right and left sides was calculated and used as an indicator of the angle of repose. The measured values are listed in Table 3 and the captured images can be found in Appendix A (Figure A1). The trend of a decrease in the angle of repose with an increase in granule diameter is present, which is consistent with previous studies [46]. The highest values of angle of repose were obtained for POM (28.74°) and CS (26.45°) granules with a diameter of 1 mm, and the lowest values were recorded for POM (17.94°) and CS (18.25°) granules with the diameter of 2 mm. Although the density of carbon steel is approximately 5.5 times higher compared to POM granules, which should result in higher angle of repose values, the obtained values are very similar.

### 3.2. Uniaxial Compression

**Figure 7:**
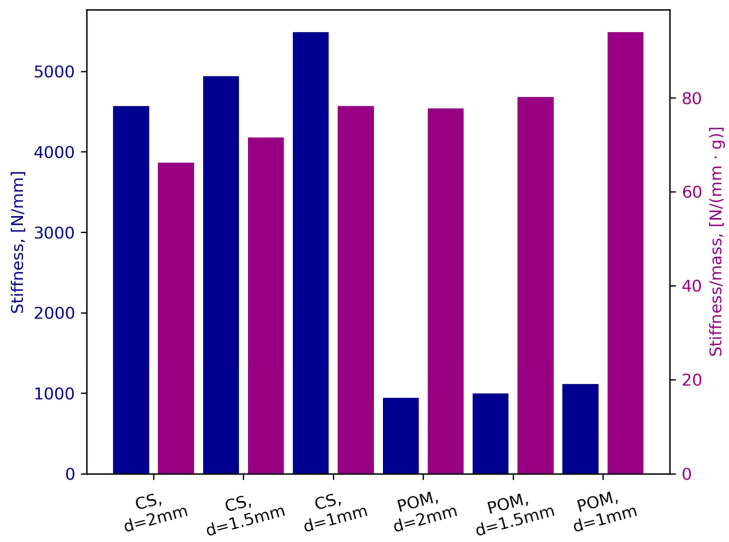
Uniaxial compression results: Stiffness (blue) and Stiffness per unit mass (purple).

**Figure A2:**
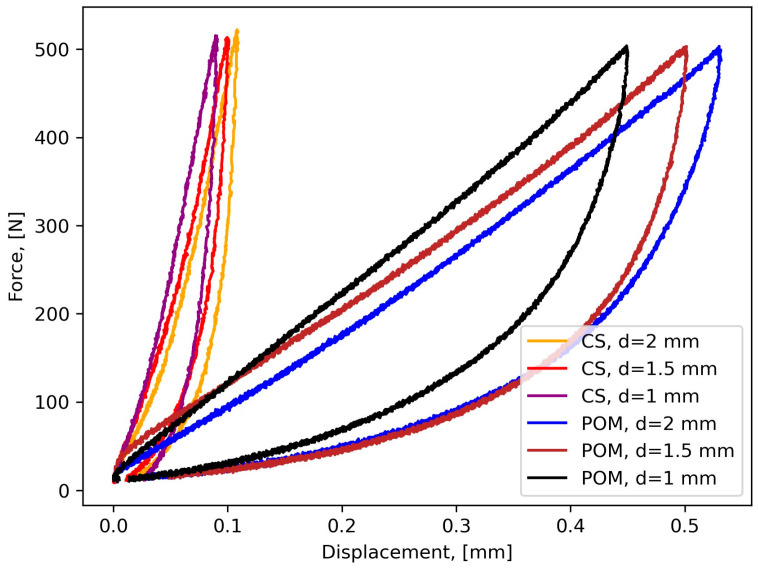
Force-displacement curves for uniaxial compression tests.

Uniaxial compression tests were conducted to comprehensively investigate the mechanical behavior of confined granular material. The main objective was to gather information on the stiffness of unconfined granular material. As stiffness represents the slope of the loading curve, it was calculated by linear regression of the last loading cycle. Additionally, considering the substantial density differences, stiffness per unit mass was calculated. The graphical representation of the results obtained during experiments is given in Figure 7, and the force–displacement curves can be found in Appendix B (Figure A2). Compared to the stiffness of POM granules, the stiffness of CS granules was up to 5 times higher. Variations in granule size within the same material showed a difference of up to 20%. Both materials exhibited an increase in stiffness with a decrease in the granule’s diameter. The lowest stiffness values were observed for 2 mm granules (4568 N/mm for CS, 940 N/mm for POM), while the highest value was recorded for 1 mm granules (5490 N/mm for CS, 1111 N/mm for POM). The stiffness of 1.5 mm granules was 4939 N/mm for CS and 996 N/mm for POM. The trend of increase in stiffness with the decrease in granule diameter continued to be present when mass was introduced. However, the advantage of CS granules disappeared. In terms of stiffness per unit mass, POM granules performed up to 20% better.

### 3.3. Damping Element Evaluation

**Figure A3:**
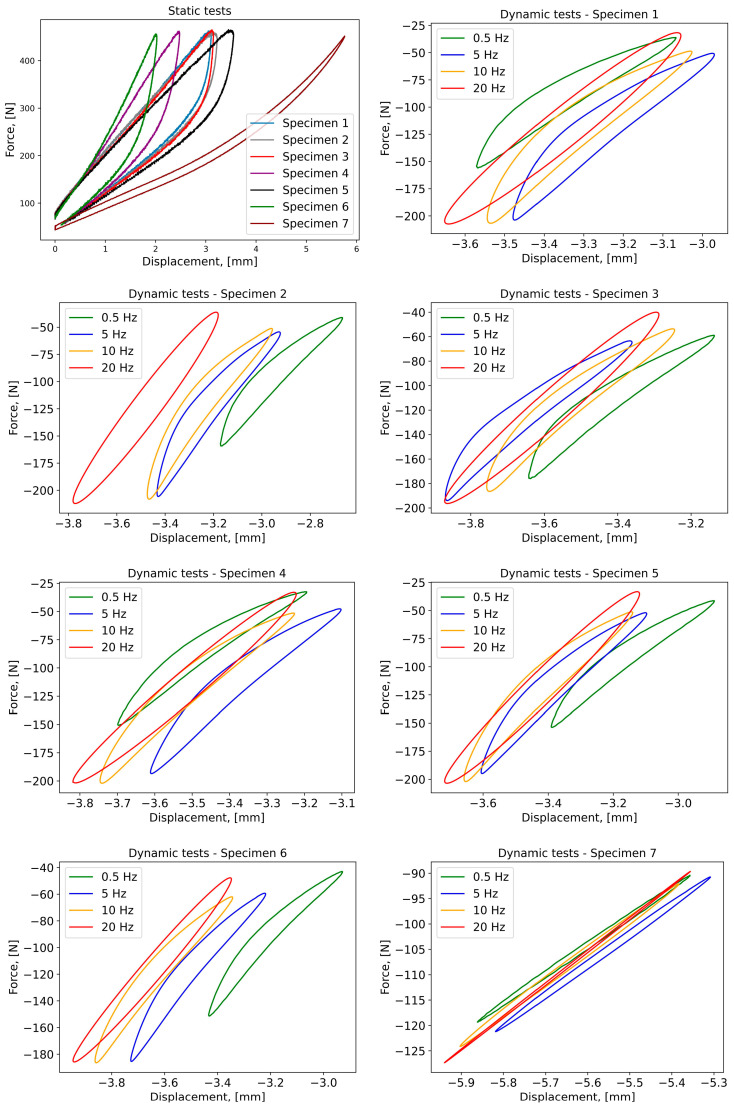
Force-displacement curves recorded for all specimens during static and dynamic tests.

The current development in vibration dampers is placing increased emphasis on incorporating additional design features such as stiffness and low mass, which must be considered for a comprehensive evaluation of vibration-damping performance. For this reason, both stiffness and damping performance, as well as the additional design parameters in which the mass was included, have been analyzed in the evaluation of damping elements. Force-displacement curves obtained during static and dynamic tests are given in Appendix C (Figure A3).

**Figure 8:**
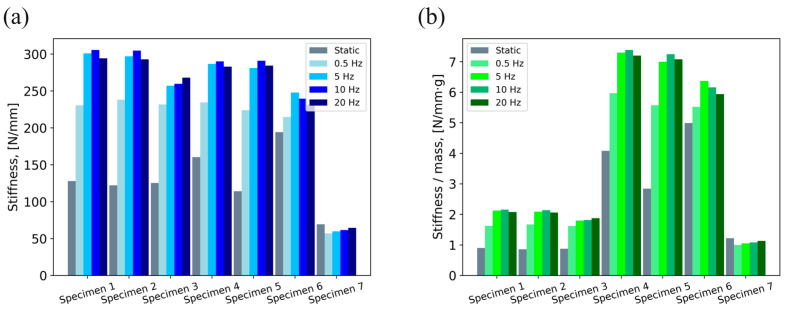
Damping element evaluation results: (**a**) stiffness, (**b**) stiffness per unit mass.

Figure 8a shows the graphical representation of the stiffness values obtained during the experiments performed on damping elements. Details on material, mass, granule diameter and volume ratio of tested specimens are given in Table 2 in Section 2.2.2. During static tests, the highest value of stiffness was recorded for Specimen 6 (194.3 N/mm) and the lowest value for Specimen 7 (69.5 N/mm). In contrast to the GDEs with POM granules (Specimens 4 to 6), GDEs with CS granules (Specimens 1 to 3) exhibited very similar stiffness values, with only a 4.7% difference between the highest (128 N/mm for Specimen 1) and the lowest value (122.3 N/mm for Specimen 2). When it comes to dynamic tests, the maximum stiffness was measured for Specimen 4 at a frequency of 10 Hz (301 N/mm), and the lowest for Specimen 7 at a frequency of 0.5 Hz (57.2 N/mm). Notably, there is a discernible trend of increasing stiffness with an increase in excitation frequency up to 10 Hz. A decrease in stiffness at a frequency of 20 Hz was recorded for Specimens 1–6. In general, the difference in stiffness at higher frequencies (5, 10 and 20 Hz) for all specimens was fairly low. Specimens 1–3 exhibited a decrease in stiffness with the decrease in granule diameter at all excitation frequencies, whereas for Specimens 4–6 this trend occurs only at lower frequencies (0.5 and 5 Hz).

The graphical representation of the results obtained for stiffness per unit mass is shown in Figure 8b. This approach significantly reduces the stiffness advantage of the GDEs with CS granules compared to the rubber DE (Specimen 7). The specific stiffness of GDEs with CS granules was lower than that of the rubber DE in the static tests, but in the dynamic tests, they exhibited up to 80% higher specific stiffness values. GDEs with POM granules performed significantly better (up to 250%) than GDEs with CS granules and the rubber DE. The highest value of specific stiffness obtained in static tests was 4.99 N/(mm∙g) for Specimen 6, and in dynamic tests 7.384 N/(mm∙g) for Specimen 4 at a frequency of 10 Hz. The lowest value in static tests was obtained for Specimen 2 (0.86 N/(mm∙g)), and in dynamic tests for Specimen 7 at a frequency of 0.5 Hz (1 N/(mm∙g)).

To quantify only the vibration-damping performance of the damping elements, the specific damping capacity (*η*) was used. Specific damping capacity (Equation (2)) is defined as the ratio of the dissipated energy ∆*U* and the maximum possible reversible energy of the system *U* [49]. Dissipated energy ∆*U* represents the area enclosed by the hysteresis loop on a force–displacement diagram.

(2) $$\eta =\frac{∆U}{U},$$

**Figure 9:**
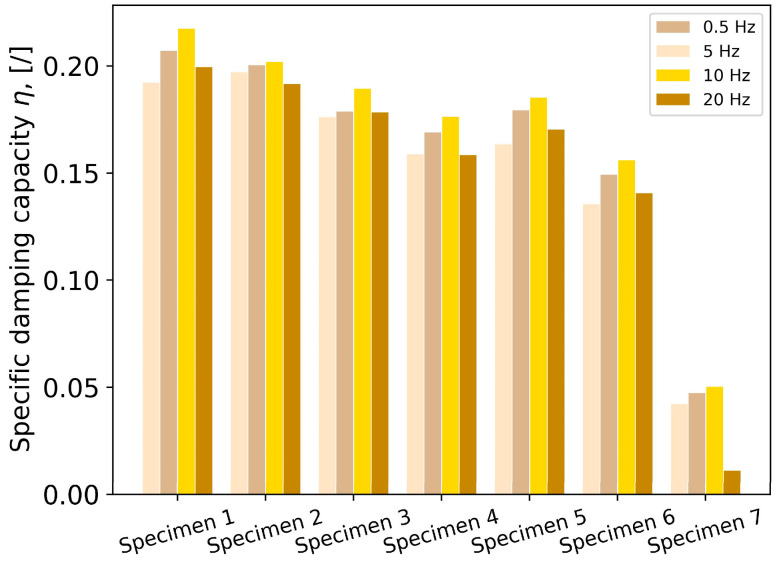
Damping performance in terms of specific damping capacity.

Specific damping capacity quantifies vibration-damping performance independently of other design features. Higher values of specific damping capacity correspond to better vibration attenuation. The results, given in Figure 9, show that the increase in excitation frequency up to 10 Hz, is accompanied by a slight increase in damping performance. At a frequency of 10 Hz, all specimens exhibited up to 15% better vibration-damping properties than at a frequency of 0.5 Hz. An average decrease of 70% in specific damping capacity for Specimen 7 was recorded at a frequency of 20 Hz, whilst for GDEs, this decrease was significantly lower, being up to 3.5%. With up to 4 times higher values of specific damping capacity at frequencies up to 10 Hz, and up to 18 times higher values for the frequency of 20 Hz, GDEs showed superior vibration-damping properties compared to conventional bulk material-based rubber damping elements. In general, GDEs exhibited similar performance regardless of the granular material, confirming that the force chain mechanism is independent of the material. The difference in average performance was up to 15% in favor of GDEs with CS granules, which can be the consequence of a higher friction coefficient in the case of CS–CS contact compared to POM–POM contact.

The highest specific damping values at all frequencies were recorded for Specimen 1 (0.1922at a frequency of 0.5 Hz, 0.207 at a frequency of 5 Hz, 0.2174 at a frequency of 10 Hz, and 0.1995 at a frequency of 20 Hz), and the lowest for Specimen 7 (0.0422 at a frequency of 0.5 Hz, 0.0473 at a frequency of 5 Hz, 0.0503 at a frequency of 10 Hz, and 0.011 at a frequency of 20 Hz). It should be noted that the second-highest values were recorded for Specimen 2 and were only up to 5% lower than the highest values. A decrease in vibration-damping performance with decreasing granule diameter was observed for CS granules, while among POM granules, the highest performance was recorded for 1.5 mm granules (Specimen 5).

The design parameter *η_k_* and the design parameter η_d_, proposed by Gosar [9], were calculated to evaluate damping performance with consideration of additional design features (stiffness and mass). The design parameter *η_k_* (Equation (3)) is defined as the ratio of the dissipated energy ∆*U* and the stiffness *k*. Additionally, the mass was included in the design parameter *η_d_* (Equation (4)).

(3) $$\eta_{k}=\frac{∆U}{k},$$

(4) $$\eta_{d}=\frac{∆U}{k\cdot m},$$

**Figure 10:**
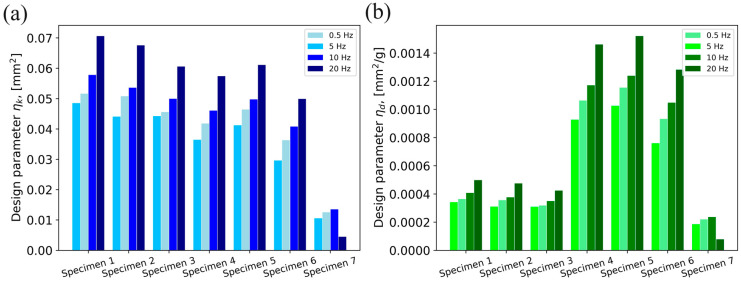
Damping performance in terms of design parameter: (**a**) *η_k_*, (**b**) *η_d_*.

Following Equations (3) and (4), the design parameters were calculated and the graphical representation of the obtained values for all specimens is given in Figure 10.

Considering only stiffness as an additional design feature, Specimens 1–3 exhibited the best performance. The maximum value of the design parameter *η_k_* of 0.0706 mm^2^ was obtained for Specimen 1 at a frequency of 20 Hz. It should be noted that the second highest value of the parameter η_k_ was recorded for Specimen 2 at a frequency of 20 Hz and was only 4% lower than the maximum value. A trend of increase in values of design parameter *η_k_* with the increase in frequencies is present. When it comes to the GDEs with POM granules, Specimen 5 performed best at all frequencies. The average difference in performance of Specimens 1–3 and Specimens 4–6 was 30%. The lowest values of the design parameter η_k_ at all frequencies were obtained for Specimen 7. GDEs performed up to 350% better than the rubber damping element at all excitation frequencies.

As expected, due to the high density of carbon steel, Specimens 1, 2 and 3 lost their advantage in performance when the mass was introduced. The variations in performance between these specimens and Specimen 7 decreased, the difference being up to 40%. The performance of Specimens 4, 5 and 6 significantly increased compared to the other specimens. The highest value of 1.52∙10^−3^ mm^2^/g of the design parameter η_d_ was obtained for Specimen 5 at a frequency of 20 Hz, which is about 3 times higher than that for Specimen 1, which performed the best among Specimens 1–3. The lowest value recorded was at a frequency of 20 Hz for Specimen 7 (7.86∙10^−5^ mm^2^/g). The difference in performance between Specimens 4, 5 and 6 is up to 16%. The lowest values of the design parameter *η_d_* among these specimens were recorded for Specimen 6 at a frequency of 0.5 Hz (7.6∙10^−4^ mm^2^/g). For all excitation frequencies, the lowest values of the design parameter η_d_ among GDEs were obtained for the specimens with the smallest granule diameter (Specimen 3 and Specimen 6).

## 4. Discussion

The main objective of this study was to compare the performance of particle dampers filled with metal (CS) and polymer (POM) granules and to gain insight into the influence of different parameters, such as flowability and granule size, on the overall damping performance. A conventional bulk material-based vibration damper was used as a reference specimen. The experimental part of this work can be divided into two phases: the first phase is the examination of granular material’s physical properties and the second phase is the evaluation of the damping elements.

In the first phase, angle of repose and uniaxial compression tests were carried out to gain insight into the flowability and uniaxial strength of unconfined material. The angle of repose tests confirmed the decrease in flowability with the decrease in granule diameter. Significant differences in the density of the used granular materials and similar angles of repose values indicate that POM granules have lower flowability compared to CS granules. Uniaxial compression tests showed that CS granules have up to 5 times higher stiffness. The increase in stiffness with decreasing granule size observed for both materials can be the consequence of a higher number of contacts per granule which can reduce the rearrangement of granules. However, the stiffness per unit mass of POM granules was up to 20% higher than that of CS granules, which indicates that POM granules have a higher stiffness-to-weight ratio.

The second phase consisted of three steps. The first step was to evaluate the stiffness of the damping elements. The results show that the geometry of the GDE container has a significant impact on stiffness, as the performance difference between GDEs with CS and GDEs with POM granules was fairly low. As expected, conventional DE performed the worst. In terms of stiffness per unit mass, GDEs with POM granules showed superior performance compared to both GDEs with CS granules and conventional DE. A trend of an increase in stiffness with an increase in excitation frequency up to 10 Hz was present. A fairly low decrease in stiffness at a frequency of 20 Hz is the result of the overshoot of the vibration amplitude, due to the high load velocities. The second step was to evaluate the damping performance in terms of specific damping capacity. GDEs with CS granules exhibited a slight advantage compared to POM granules. The increase in damping performance with increasing granule size and test frequency (up to 10 Hz) is present. A decrease in specific damping capacity at a frequency of 20 Hz was present for all specimens, and it is a consequence of higher forces that increase the amount of total reversible energy U. In the third step, the damping performance was evaluated with the inclusion of design features (stiffness and mass). The introduction of stiffness did not result in significant changes in performance, while GDEs with POM granules exhibited a superior performance when mass was introduced due to the advantage of a higher stiffness-per-weight ratio of POM.

## 5. Conclusions

The proper integration of granular materials within a system has been proven to offer dual benefits: enhancing vibration-damping properties and increasing the load-bearing capacity of structures. This improvement is achieved by utilizing the unique energy dissipation mechanism of granular damping elements, which is primarily based on the formation of force chains rather than the internal damping of materials as observed in conventional dampers. This particular property of granular damping elements offers an opportunity for better waste management, particularly for materials such as plastic and rubber waste. The effective use of such materials in the production of granular damping elements can significantly reduce their environmental impact. Also, the use of low-price materials such as sand is possible since the force chain mechanism as shown is independent of the material.

Although the results show that the main mechanism of energy dissipation is not related to the internal damping of the material, the choice of granular material still has a significant impact on the overall performance of particle dampers, especially if the mass of the damping element is important. This is demonstrated by the superior performance of POM granules compared to CS granules in terms of design parameters η_k_ and η_d_, where stiffness and mass were considered in addition to vibration damping.

When it comes to the granule parameters, the granule size and the flowability showed the greatest influence on the damping performance of GDEs. The influence of these parameters comes from the close correlation to particle rearrangements and force chain formation. The geometry of the GDE container can be used to tune the stiffness of GDEs which can be very important to avoid the occurrence of natural frequencies in the operating range. Another highly influencing parameter is prestress, previously discussed in [20,22,23,24,25,26]. All these parameters can be varied to tune the granular damping properties and optimize its vibration-damping and load-bearing performance.

Although this paper has provided valuable insights into the behavior of granular damping elements, further experimental investigations, which can include different combinations of previously mentioned influencing parameters, a broad range of test frequencies, and numerical simulations, are required for a deeper understanding of the dissipation mechanisms and the production of optimized, cost-effective and high-performance load-bearing granular damping elements.

## Figures and Tables

**Table 1:** Physical properties of granular materials.

Physical Property	Unit	Carbon Steel	POM
Young’s modulus (E)	GPa	(200–210)	(2.5–3.5)
Poisson’s ratio (ν)	/	(0.29–0.33)	(0.36–0.4)
Density (ρ)	kg/m^3^	7850	1420

**Table 2:** Details on material, mass, granule diameter and volume ratio of fabricated specimens.

Spec.	Material	Mass [g]	Diameter [mm]	Volume Ratio [/]
1	Carbon steel	141.74	2.0	0.531
2	Carbon steel	142.35	1.5	0.552
3	Carbon steel	142.97	1.0	0.521
4	POM	39.30	2.0	0.579
5	POM	40.17	1.5	0.582
6	POM	38.91	1.0	0.585
7	NR + SBR	57.01	/	/

**Table 3:** Angle of repose—experiment results.

Granular Material	Granule Diameter [mm]	Angle of Repose [°]
CS	2.0	18.25
CS	1.5	21.20
CS	1.0	26.45
POM	2.0	17.21
POM	1.5	19.75
POM	1.0	28.45

## Data Availability

Data available on request due to the ongoing research.
